# Study on the Effect of Huanglian Jiedu Decoction on the Composition of Gut Microflora in SD Rats Based on 16S rRNA Sequencing

**DOI:** 10.1155/2020/8872439

**Published:** 2020-12-08

**Authors:** Haiyang Du, Gengxin Zhang, Guangyong Yang, Weiyi Tian, Wenjia Wang, Ping Wang, Guangzhi He

**Affiliations:** College of Basic Medicine, Guizhou University of Traditional Chinese Medicine, Guiyang 550025, China

## Abstract

**Objective:**

To study the effects of Huanglian Jiedu Decoction (HJD) on gut microflora of SD rats.

**Method:**

After 3 days of adaptive feeding, 36 rats were randomly divided into 4 groups, namely, the normal control group (NC, 10 mL/kg, distilled water), high_HJD dose group (H_HJD, 6.25 g/kg, weight ratio between crude drug and rat), medium_HJD dose group (M_HJD, 3.125 g/kg), and low_HJD dose group (L_HJD, 1.56 g/kg), and each group consisted of 9 mice. The HJD groups were then treated with orally administered HJD for 21 days, while the NC group was treated with distilled water. After 7 days, 14 days, and 21 days of the experiment and after 12 hours of fasting and water deprivation, 3 SD rats in each group were randomly sacrificed by cervical dislocation and sterile operation to collect stool faces. Sample DNA was extracted by Fecal total DNA extraction kit, sequenced using Illumina MiSeq platform, and analyzed.

**Results:**

The abundance of *Romboutsia*, *Turicibacter*, *Lactobacillus*, and *Gemella* decreased, while that of *Escherichia_Shigella*, *Coprobacillus*, *Blautia, Akkermansia*, *Klebsiella, Rhodococcus*, *Parabacteroides*, *Citrobacter*, *Enterococcus*, *Bacteroides*, and *Erysipelotrichaceae_incertae_sedis* increased after using H_HJD. The abundance of *Gemella, Turicibacter*, *Romboutsia*, and *Lactobacillus* decreased, while that of *Blautia*, *Akkermansia*, Escherichia_Shigella, *Thiobacillus*, *Rothia*, *Enterococcus*, *Lactobacillus*, and *Erysipelotrichaceae-incertae-sedis* increased after using M_HJD. The abundance of *Romboutsia*, *Turicibacter*, *Lactobacillus*, and *Gemella* decreased, while that of Escherichia_Shigella, *Bacteroides*, *Akkermansia*, *Enterococcus*, *Rhodococcus*, *Parabacteroides*, *Desulfovibrio*, *Blautia*, *Fusobacterium*, *Rothia*, and *Streptococcus* increased after using L_HJD.

**Conclusion:**

HJD can regulate gut microbiota, and its effect varies with different dosage and duration of medication.

## 1. Introduction

As the normal microbiota in the intestine, gut microbiota not only plays an important role in the physiological functions of the intestine but also plays an important role in the host's physiology, metabolism, immunity, digestion, and nutrition uptake [1]. And the composition of gut microbiota is altered in many disease states, including type 2 diabetes mellitus (T2DM), high blood pressure (BP), colitis, cardiovascular disease, and obesity [2]. Dysbiosis in the gut microbiota of type 2 diabetes mellitus (T2DM) patients is characterized by a higher proportion of *Bacteroides* [3]. Li and his colleagues found that the gut microbiota in patients with hypertension had a higher percentage of an enterotype rich in bacteria from the genus *Prevotella* [4]. In the gut microbiota of colitis patients, there was imbalance in Bacteroidetes, and species diversity decreased [5]. The stable balance between host and gut microflora plays an indispensable role in maintaining human health and has become a research object of widespread concern. Huanglian Jiedu Decoction (HJD), first recorded in the book “Wai-Tai-Mi-Yao” in the Tang dynasty, is a representative agent for clearing away heat and detoxification in traditional Chinese medicine [6]. It has been clinically practiced in the treatment of T2DM, cerebrovascular disease , hypertension, cancer, and other diseases in China. [7–11]. However, the mechanism of HJD for the treatment of diseases is currently lacking. Exploring the role and mechanism of traditional Chinese medicine through the gut microflora has become a research hotspot in the treatment of disease [12].

Therefore, 16S rRNA sequencing technology was used to analyze the effect of HJD on the structure of gut microflora in SD rats and to clarify the effect of HJD on gut microflora and provide reference for clinical application.

## 2. Methods

### 2.1. Preparation of HJD

The traditional Chinese medicine (TCM) formula in our study was HJD, composed of four herbs, namely, *Rhizoma Coptidis*, *Radix Scutellariae*, *Phellodendron amurense* Rupr, and *Gardenia jasminoides* Ellis. Herbs were purchased from Beijing Tongrentang Guiyang Branch. We prepared HJD referred to the optimized method described by Guang Chen [13]. Details are as follows: distilled water was used for the preparation of samples. *Rhizoma Coptidis* (9 g), *Radix Scutellariae* (6 g), *Phellodendron amurense* Rupr (6 g), and *Gardenia jasminoides* Ellis (9 g) were crushed into small pieces with scissors and mixed. The mixture was soaked in 1000 mL of cold water for 40 min before being boiled for 1.5 h. Filtrates were collected, and the residues were then boiled with an addition of 300 mL water for 40 min. Finally, the two filtrates were combined and concentrated to a ﬁnal volume of 48 mL (0.625 g/mL) by rotary evaporation instrument (model: RE5203, Shanghai Yarong biochemical instrument factory). The filtrate was stored in seal at 4°C.

### 2.2. Animal Experiments

#### 2.2.1. Animals

Specific-pathogen-free (SPF) Sprague Dawley rats were purchased from Chongqing Tengxin Biotechnology Co., Ltd. (age, 20 ± 2 d, weight, 200 ± 20 g, 50% female; *n*, 36). Rats were acclimatized to the laboratory for 3 days prior to the experiments.

#### 2.2.2. Experimental Processes

The animal experiments were conducted by referencing the methods of a similar study with minor modiﬁcations [14]. We randomly divided the 36 rats into 4 groups, namely, the normal control group (NC, 10 mL/kg, distilled water), high_HJD dose group (H_HJD, 6.25 g/kg, weight ratio between crude drug and rat), medium_HJD dose group (M_HJD, 3.125 g/kg), low_HJD dose group (L_HJD, 1.56 g/kg), and each group consisted of 9 mice. The HJD groups were then treated with orally administered HJD for 21 days, while the NC group was treated with distilled water. After 7 days, 14 days, and 21 days of the experiment, after 12 hours of fasting and water deprivation, 3 SD rats in each group were randomly sacrificed by cervical dislocation for aseptic operation to collect faeces. Faeces were frozen immediately with liquid nitrogen after sampling and stored at −80°C (model: DW-86L490 J, Qingdao Haier biomedical Co., Ltd.) until the DNA extraction.

### 2.3. Analysis of Fecal 16S rRNA

The composition of gut microbiota was detected by 16S rRNA sequencing analysis. Microbial genomic DNA was extracted from fecal samples using a fecal total DNA extraction kit (batch number: DP328-2, Tiangen Beijing Biochemical Technology Co., Ltd.) according to the manufacturer's protocols. The extracted DNA from each fecal sample was ampliﬁed at the V4 region of the 16S rRNA genes by using speciﬁc primers: 338F (5′'-ACTCCTACGGGAGGCAGCA-3′') and 806R (5′'- GGACTACHVGGGTWTCTAAT-3′'). The qualiﬁed amplicons were pooled into equimolar fractions and paired-end sequenced (PE250) on an Illumina MiSeq platform (Shanghai Biotree Biotech Co., Ltd, Shanghai, China).

### 2.4. Bioinformatics Analysis

Reads were assembled and analyzed using Vsearch (version 2.14.1) and Usearch10 [15, 16]. After quality filtering and chimera removal, clean sequences and exact amplicon sequence variants (ASVs) were resolved using Unoise3. Taxonomy was assigned to ASVs using Usearch10 -sintax with -strand_both and -sintax_cutoff 0.6. ASV abundance was normalized using a standard of sequence number corresponding to the sample with the least sequences. Subsequent analysis of alpha-diversity and beta-diversity were all performed basing on these output normalized data. Linear discriminant analysis effect size (LEfSe) was performed to find biomarker(s) differentially represented between NC and HJD groups [17]. PICRUSt2 (Phylogenetic Investigation of Communities by Reconstruction of Unobserved States 2) is used to impute MetaCyc pathway abundances from the original microbial abundance data [18]. And NC group data were used in our previous paper [19].

## 3. Results

### 3.1. Bacterial Alpha- and Beta-Diversity

**Figure 1:**
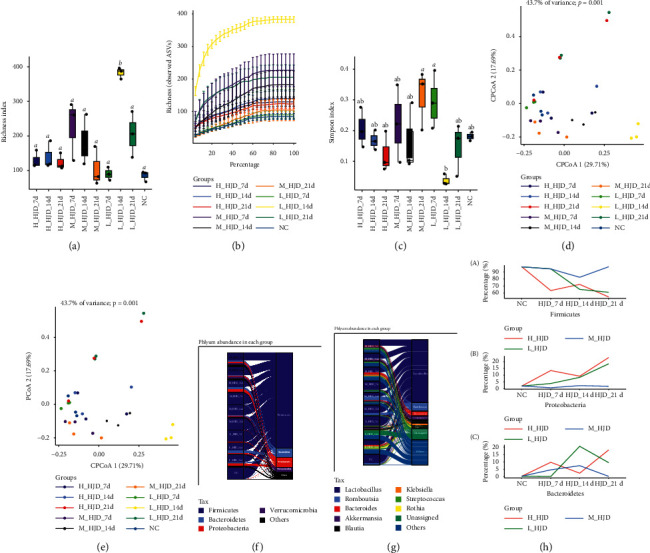
Gut microbiota diversity and community composition after HJD administration: (a) rarefaction curves of detected bacterial species of the microbiota reach the saturation stage with increasing numbers of samples, indicating that the gut microbiota in our population capture most intestinal bacteria members from different dose and time of HJD and NC. Each vertical bar represents standard error. The numbers of replicated samples in this figure are as follows: in H_HJD, 7d (*n* = 3), 14d (*n* = 3), and 21d (*n* = 3), in M_HJD, 7d (*n* = 3), 14d (*n* = 3), and 21d (*n* = 3), in L_HJD, 7d (*n* = 3), 14d (*n* = 3), and 21d (*n* = 3), in NC (*n* = 3). The following table and figure are the same. (b), (c) Richness and Shannon index of gut microbiota from different dose and time of HJD and NC. The horizontal bars within boxes represent medians. The tops and bottoms of boxes represent the 75th and 25th percentiles, respectively. The upper and lower whiskers extend to data no more than 1.5x  the interquartile range from the upper edge and lower edge of the box, respectively. The tops of boxes sign lowercase letter completely different, indicated difference remarkable (*P* > 0.05), and it has the same lowercase letter or the nonletter expression difference which is not remarkable (*P* > 0.05). (d) Unconstrained PCoA (for principal coordinates PCo1 and PCo2) with Bray–Curtis distance showing that the gut microbiota of SD rats from different dose and time of HJD and NC. (e) Constrained principal coordinate analysis with Bray–Curtis distance showing that the gut microbiota of HJD and NC. (f), (g) The relative abundance of gut microbiome at the phylum and genus level corresponding to different groups is shown, and the width of branches indicates the relative abundance of species. (h) The line chart shows the changes of the abundance of Firmicutes, Proteobacteria, and Bacteroidetes at different times and groups. Note. H_HJD_7d: H_HJD on day 7; H_HJD_14d: H_HJD on day 14; H_HJD_21d: H_HJD on day 21; M_HJD_7d: M_HJD on day 7; M_HJD_14d: M_HJD on day 14; M_HJD_21d: M_HJD on day 21; L_HJD_7d: L_HJD on day 7; L_HJD_14d: L_HJD on day 14; L_HJD_21d: L_HJD on day 21; NC: three rats were randomly selected from the NC group.

Rarefaction analysis revealed that our population captured most gut microbiota members from each SD rat. Measurement of within-sample diversity (*α*-diversity) revealed a significant difference between HJD and NC varieties (Figures 1(b) and 1(c)). The gut microbiota of L_HJD_14d had higher abundance than NC (Figure 1(b)), indicating that taking low-dose HJD for 14d can increase species richness. The gut microbiota of L_HJD_7d, M_HJD_21d had higher diversity than NC (Figure 1(c)), indicating that taking low-dose HJD for 7d and L_HJD_7d can increase species diversity. It is interesting that the species diversity of L_HJD_14d was lower than that of NC. Unconstrained principal coordinate analysis (PCoA) of Bray–Curtis distance revealed that the gut microbiota of different dose and time of HJD and NC formed distinct clusters, in which the separation was not well along the first coordinate axis. The constrained principal coordinate analysis with Bray–Curtis distance showing that the contribution rate of current conditions to the total difference among samples was 43.7.2%, and there was significant difference among groups (*P* < 0.01).

### 3.2. Gut Microflora Structure Analysis

Next, we further analyzed the relative abundances of differential bacteria in the gut microbiota at phylum and genus levels. At the phylum level, phylum Firmicutes, Proteobacteria, and Bacteroidetes were most abundantly presented which accounted for the majority of the population in all groups (Figure 1(f)), and at the genus level, *Lactobacillus* and *Romboutsia* were most abundantly presented which accounted for the majority of the population in most groups (Figure 1(g)). The abundance of Firmicutes, Proteobacteria, and Bacteroidetes changes in different groups over time (Figure 1(h)). We can find that no matter which bacteria, the final abundance of the M_HJD tends to be normal.

To identify specific bacterial taxa associated with HJD, we compared gut microbiota using LEfSe, and the criteria were set at a linear discriminant analysis (LDA) ＞ 2. In the first week, at the phylum level, compared with NC, the abundance of Firmicutes was significantly reduced in H_HJD_7d and the abundance of Verrucomicrobia and Bacteroidetes was significantly increased; at the genus level, the abundance of *Romboutsia*, *Turicibacte*r, and *Clostridium sensu stricto* was significantly reduced and the abundance of *Escherichia*-*Shigella*, *Coprobacillus*, *Enterococcus*, *Blautia, Bacteroides*, and *Akkermansia* increased significantly in H_HJD_7d (Figure S1).

Compared with NC, at the phylum level, Proteobacteria significantly decreased and Verrucomicrobia increased significantly in M_HJD_7d; at the genus level, the abundances of *Gemella*, *Clostridium_sensu_stricto*, *Turicibacter,* and *Romboutsia* decreased significantly and the abundances of *Blautia*, *Akkermansia*, *Escherichia*-*Shigella*, and *Thiobacillus* increased significantly in M_HJD_7d (Figure S2).

Compared with NC, L_HJD_7d has no significant difference at the phylum level; at the genus level, the abundances of *Romboutsia*, *Turicibacte*r, and *Clostridium_sensu_stricto* were significantly reduced and the abundances of *Escherichia*-*Shigella*, *Bacteroides*, *Akkermansia*, and *Enterococcus* were significantly increased in L_HJD_7d (Figure S3).

In the second week, at the phylum level, compared with NC, the abundance of Firmicutes was significantly reduced, and the abundances of Fusobacteria, Verrucomicrobia, and Bacteroidetes were significantly increased in H_HJD_14d; at the genus level, the abundances of *Lactobacillus*, *Clostridium_sensu_stricto*, and *Turicibacter* were significantly decreased and the abundances of *Coprobacillus*, *Escherichia*-*Shigella*, *Klebsiella*, *Bacteroides*, *Fusobacterium*, *Enterococcus*, *Blautia*, and *Akkermansia* increased significantly in H_HJD_14d (Figure S4).

Compared with NC, at the phylum level, the abundance of Actinobacteria was significantly reduced, and the abundances of Verrucomicrobia and Bacteroidetes were significantly increased in M_HJD_14d; at the genus level, the abundances of *Rothia*, *Gemell*a, and *Lactobacillu*s were significantly reduced and *Akkermansia*, *Bacteroides, Blautia*, *Escherichia*-*Shigella*, *Clostridium_XlVa*, *Erysipelotrichaceae_incertae_sedis*, *Enterococcus*, and *Clostridium_XVIII* increased significantly in M_HJD_14d (Figure S5).

Compared with NC, at the phylum level, the abundance of Firmicutes was significantly reduced and the abundances of Verrucomicrobia and Bacteroidetes were significantly increased in L_HJD_14d; at the genus level, the abundances of *Lactobacillus*, *Romboutsia*, *Rhodococcus,* and *Gemella* were significantly reduced and the abundances of *Prevotella*, *Clostridium_XlVa*, *Akkermansia*, *Parabacteroides*, *Escherichia*-*Shigell*a, *Eubacterium*, *Bacteroides*, *Desulfovibrio*, and *Blautia* increased significantly in L_HJD_14d (Figure S6).

In the third week, at the phylum level, compared with NC, the abundance of Firmicutes was significantly reduced and the abundances of Verrucomicrobia, Proteobacteria, and Bacteroidetes were significantly increased in H_HJD_21d; at the genus level, the abundances o*f Romboutsia*, *Turicibacter*, *Clostridium_sensu_stricto*, and *Gemella* were significantly reduced and the abundance of *Clostridium_XVIII*, *Erysipelotrichaceae_incertae_sedis*, *Parabacteroides*, *Citrobacter*, *Enterococcu*s, *Coprobacillus*, *Eubacterium*, *Escherichia*_*Shigella*, *Akkermansia*, *Blautia*, *Klebsiella*, and *Bacteroides* increased significantly in H_HJD_21d (Figure S7).

Compared with NC, M_HJD_21d has no significant difference at the phylum level; at the genus level, the abundance of *Gemella* is significantly reduced and the abundance of *Enterococcus* is significantly increased M_HJD_21d (Figure S8).

At the phylum level, the abundance of Firmicutes decreased significantly and the abundances of Fusobacteria, Bacteroidetes, and Actinobacteria increased significantly in L_HJD_21d; at the genus level, the abundances of *Lactobacillus*, *Romboutsia*, and *Turicibacter* decreased significantly and the abundances of *Blautia*, *Fusobacterium*, *Rhodococcus*, *Escherichia*_*Shigella Enterococcus*, *Bacteroides*, *Clostridium_XlVa*, *Rothia*, and *Streptococcus* increased significantly in L_HJD_21d (Figure S9).

### 3.3. Metabolic Pathway Predictions

**Figure 2:**
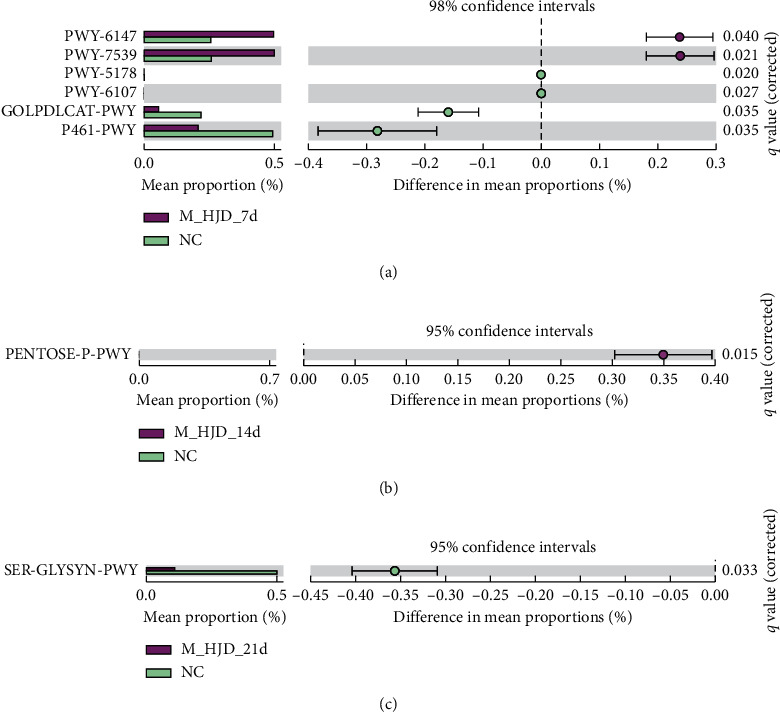
Gut microbiota metabolic pathway changes after M_HJD administration: (a) M_HJD_7d vs NC. (b) M_HJD_14d vs NC. (c) M_HJD_21d vs NC. Extended error bar plot to determine the significant difference in the number of metabolites in each group. The right side of the zero-point indicates the larger effect on the gene function in the M_HJD-NC, and the left side of the zero-point indicates the negative effect of that in the NC group. *P* value at the side indicates the significance levels between the upper and lower bars. Differences were assessed by Welch's *t*-test for two groups with Benjamini–Hochberg *P* value correction for multiple hypothesis testing.

To determine whether the observed taxonomic shifts in gut microbiota might alter their functional capabilities, we used PICRUSt2 to infer genomic content combined with STAMP to determine whether functions were differentially abundant. A total of 8 MetaCyc pathways were generated using the composition of the gut microbiota based on PICRUSt2 in M_HJD versus NC (Figure 2). PWY-6147 (6-hydroxymethyl-dihydropterin diphosphate biosynthesis I) and PWY-7539 (6-hydroxymethyl-dihydropterin diphosphate biosynthesis III) were increased in M_HJD_7d (Figure 2(a)). PENTOSE-P-PWY (pentose phosphate pathway) was increased in M_HJD_14d (Figure 2(b)). SER-GLYSYN-PWY (superpathway of L-serine and glycine biosynthesis I) decreased in M_HJD_21d (Figure 2(c)).

## 4. Discussion

TCM treatment of diseases emphasizes “the coordination of the five zang-viscera and the unity of man and nature,” and its views are highly similar to the system theory and balance theory of microecology [20]. Syndromes have the same treatment and different treatments are different in treatment based on syndrome differentiation. For this reason, the same prescription can treat different diseases. In recent years, the mechanism of action of TCM through the gut microflora has been widely recognized with the continuous in-depth research on the gut microbiota.

The work explored the effect of HJD on gut microbiota from different doses, the results show that the abundance of *Romboutsia, Turicibacter*, *Lactobacillus*, and *Gemella* decreased, while that of *Escherichia*_*Shigella*, *Coprobacillus*, *Blautia, Akkermansia*, *Klebsiella*, *Rhodococcus*, *Parabacteroides*, *Citrobacter*, *Enterococcus*, *Bacteroides*, and *Erysipelotrichaceae_incertae_sedis* increased after using H_HJD. The abundance of *Gemella, Turicibacter*, *Romboutsia*, and *Lactobacillus* decreased, while that of *Eshibacter_ShigellaBlautia*, *Akkermansia*, *Thiobacillus*, *Rothia*, *Enterococcus*, *Lactobacillus*, and *Erysipelotrichaceae_incertae_sedis* increased after using M_HJD. The abundance of *Romboutsia*, *Turicibacter*, *Lactobacillus*, and *Gemella* decreased, while that of *Eshibella_Shigella*, *Bacteroides*, *Akkermansia*, *Enterococcus*, *Rhodococcus*, *Parabacteroides*, *Desulfovibrio*, *Blautia*, *Fusobacterium*, *Rothia*, and *Streptococcus* increased after using L_HJD.

Although different doses of HJD have different effects on gut microflora of SD rats, there are still some of the same effects on *Romboutsia*, *Turicibacter*, *Escherichia coli-Shigella*, *Braute, Akkermansia, Lactobacillus, Gemella,* and *Enterococcus.* Studies have confirmed that the abundance of *Akkermansia* and *Braute* in diabetic patients has significantly decreased, while the abundance of *Lactobacillus* has increased [21]. This may be one of the mechanisms of HJD in treating diabetes.

Academician Wang Yongyan proved through experiments that HJD has a good preventive effect on Alzheimer's disease [22]. Ketogenic diet can effectively reduce the risk of Alzheimer's disease by increasing the abundance of *Akkermansia* and *Lactobacillus* and reducing the abundance of *Turicibacter* [23]. In this study, different doses can positively regulate *Akkermansia*, *Braute*, and *Lactobacillus*, suggesting that HJD may be effective in preventing diabetes and Alzheimer's disease by acting on this type of bacteria.

HJD also has a certain preventive effect on colorectal cancer [24–26]. Metagenomic analysis shows that *Gemella* is significantly increased in the gut microbiota of patients with colorectal cancer [27]. Qin Huanlong's research team showed that the abundance of *Escherichia-Shigella* in the intestine of sporadic colorectal cancer was significantly decreased [28]. The results of this experiment show that after the intervention of HJD, *Gemella* was significantly decreased, and *Escherichia-Shigella* was significantly increased, which further suggested the application value of HJD in the prevention and treatment of colorectal cancer.

### 4.1. Analysis from the Specificity of Each Dose Group

*Coprobacillus*, *Klebsiella*, and *Citrobacter* were only significantly affected in the H_HJD. *Thiobacillus* was only significantly affected in the M_HJD. *Streptococcus*, *Fusobacterium*, and *Desulfovibrio* were only significantly affected in the L_HJD.

Studies have shown that the abundance of *Coprobacillus*, in patients with acne, is significantly reduced, and it has been reported that HJD can treat acne [29, 30]. The abundance of *Coprobacillus* increased in H-HJD that explained part of the mechanism of HJD in the treatment of acne from the gut microbiota.

Bad breath is generally produced by heat evil in the stomach and intestines, and its main odor comes from sulfide [31]. *Thiobacillus* can oxidize sulfide (H_2_S) to sulfate [32]. The increase of *Thiobacillus* in the middle-dose group suggests that *Thiobacillus* may be one of the targets of HJD in the treatment of bad breath.

### 4.2. Analysis from the Duration of Medication

For *Akkermansia*, the significant differences in the M_HJD_21d and L_HJD_21d groups disappeared, suggesting that H_HJD is needed to improve the abundance of *Akkermansia* for a long time.

For *Blautia*, it only began to increase significantly at 14 days in the L_HJD group, suggesting that *Blautia* needs a cumulative effect.

However, in the 7-day period of *Lactobacillus,* there is no significant difference in each dose group, suggesting that HJD has little correlation with its effect and dose but is related to the duration of medication.

Studies have shown that *Erysipelotrichaceae_incertae_sedis* is a beneficial bacteria, and its abundance is negatively correlated with the blood sugar level, insulin level, and fat mass of female offspring [33]. The increase in the abundance of the bacteria in the H_HJD_14d and H_HJD_21d groups in this experiment further shows that HJD has a therapeutic effect on diabetes, but it requires 14 days of medication.

## 5. Conclusions

To sum up, this study comprehensively studied the effect of HJD on gut microbiota from different doses and different administration time. At the same time, it expounds some potential target bacteria of HJD in the prevention and treatment of diabetes, Alzheimer's disease, acne, bad breath, and colorectal cancer, but its causality needs to be further studied.

## Data Availability

The sequence data used to support the findings of this study have been deposited to the NCBI Sequence Read Archive under the BioProject accession number PRJNA664442.

## Supplementary Materials

Supplementary Materials Figure S1: lefse analysis result figure of HJD and NC group.
