# Supplementary material for: Study on the Effect of Huanglian Jiedu Decoction on the Composition of Gut Microflora in SD Rats Based on 16S rRNA Sequencing
Source: Evid Based Complement Alternat Med. 2020 Dec 8;2020:8872439. doi: 10.1155/2020/8872439 (PMC7738789; doi:10.1155/2020/8872439)
Supplement: Supplementary Materials — Figure S1: lefse analysis result figure of HJD and NC group. [file 8872439.f1.pdf]

Study on the Effect of Huanglian Jiedu Decoction on the composition of Gut Microflora in SD Rats Based on 16S rRNA Sequencing

Du Haiyang[1]; Yang Guangyong[1]; Zhang Gengxin[1]; Tian Weiyi[1]; Wang Wenjia[1]; Wang Ping[1]; He Guangzhi*

1.Guizhou University of Traditional Chinese Medicine, College of Basic Medicine，Guiyang (550025), Guizhou，China

*Corresponding Author

E-mail addresses:

He Guangzhi, E-mail: heguangzhi436@gzy.edu.cn

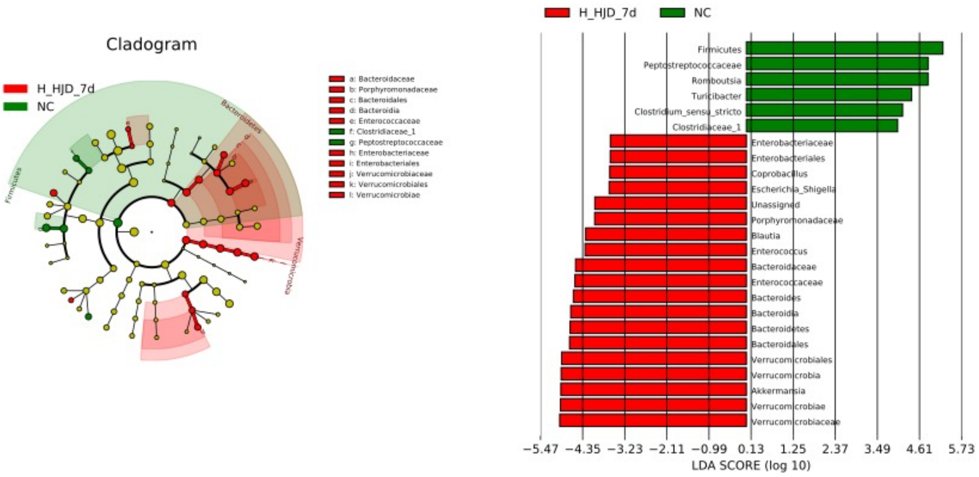

Figure S1

Taxonomic cladogram obtained by LEfSe. Differences are represented by the color of the most abundant class. Histogram of the linear discriminant analysis (LDA) score for the gut microbiota in the NC and H_HJD_7d of rats. The statistically significant differences in the microbiota of the two groups were analyzed using linear discriminant analysis effect size (LEfSe) statistical analysis for the identification of biomarkers between two groups and is shown in the histogram. The length of histogram represents the degree of influence of the different microbiota. Red indicating H_HJD_7d, green NC.

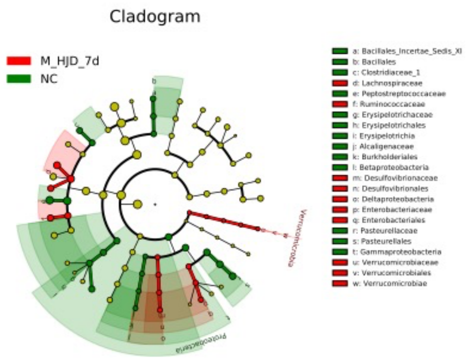

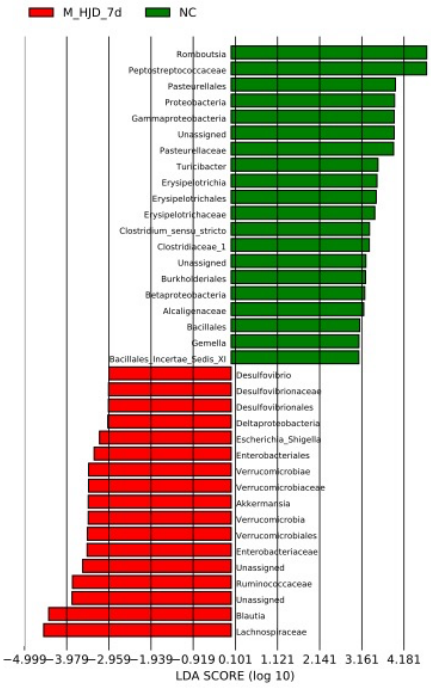

Figure S2

Taxonomic cladogram obtained by LEfSe. Differences are represented by the color of the most abundant class. Histogram of the linear discriminant analysis (LDA) score for the gut microbiota in the NC and M_HJD_7d of rats. The statistically significant differences in the microbiota of the two groups were analyzed using linear discriminant analysis effect size (LEfSe) statistical analysis for the identification of biomarkers between two groups and is shown in the histogram. The length of histogram represents the degree of influence of the different microbiota. Red indicatingM_HJD_7d, green NC.

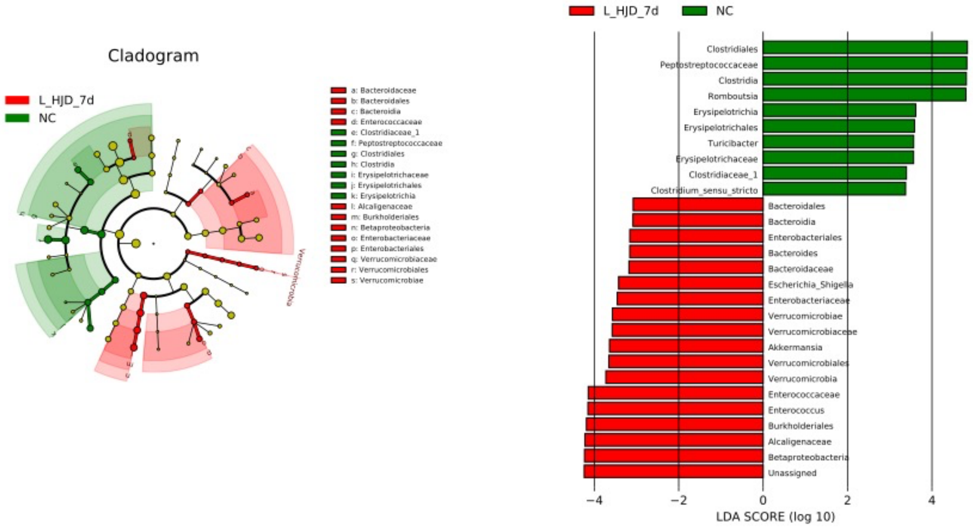

Figure S3

Taxonomic cladogram obtained by LEfSe. Differences are represented by the color of the most abundant class. Histogram of the linear discriminant analysis (LDA) score for the gut microbiota in the NC and L_HJD_7d of rats. The statistically significant differences in the microbiota of the two groups were analyzed using linear discriminant analysis effect size (LEfSe) statistical analysis for the identification of biomarkers between two groups and is shown in the histogram. The length of histogram represents the degree of influence of the different microbiota. Red indicating L_HJD_7d, green NC.

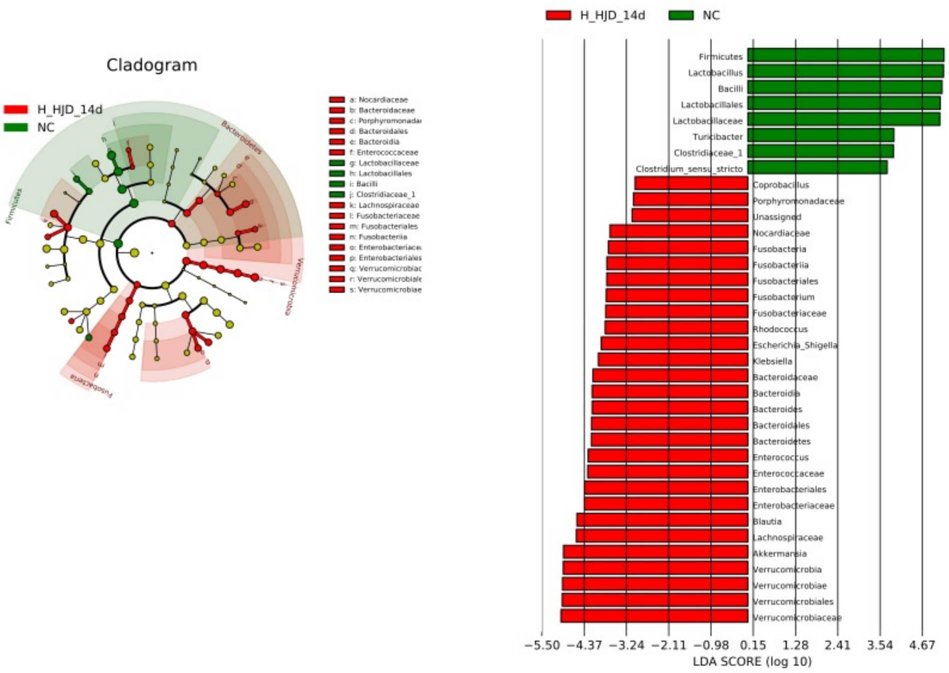

Figure S4

Taxonomic cladogram obtained by LEfSe. Differences are represented by the color of the most abundant class. Histogram of the linear discriminant analysis (LDA) score for the gut microbiota in the NC and H_HJD_14d of rats. The statistically significant differences in the microbiota of the two groups were analyzed using linear discriminant analysis effect size (LEfSe) statistical analysis for the identification of biomarkers between two groups and is shown in the histogram. The length of histogram represents the degree of influence of the different microbiota. Red indicating H_HJD_14d, green NC.

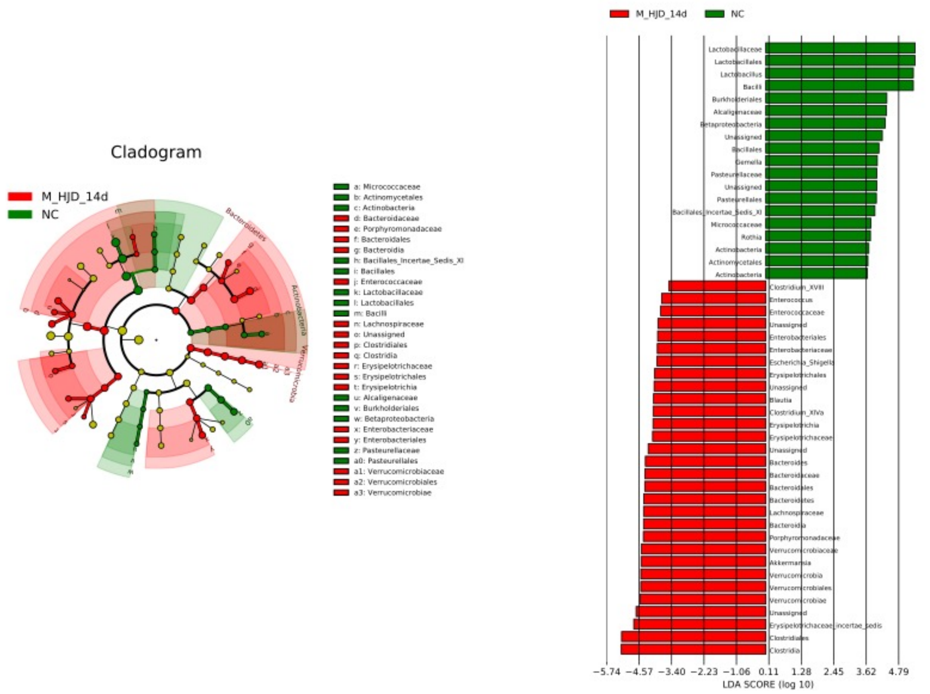

Figure S5

Taxonomic cladogram obtained by LEfSe. Differences are represented by the color of the most abundant class. Histogram of the linear discriminant analysis (LDA) score for the gut microbiota in the NC and M_HJD_14d of rats. The statistically significant differences in the microbiota of the two groups were analyzed using linear discriminant analysis effect size (LEfSe) statistical analysis for the identification of biomarkers between two groups and is shown in the histogram. The length of histogram represents the degree of influence of the different microbiota. Red indicating M_HJD_14d, green NC.

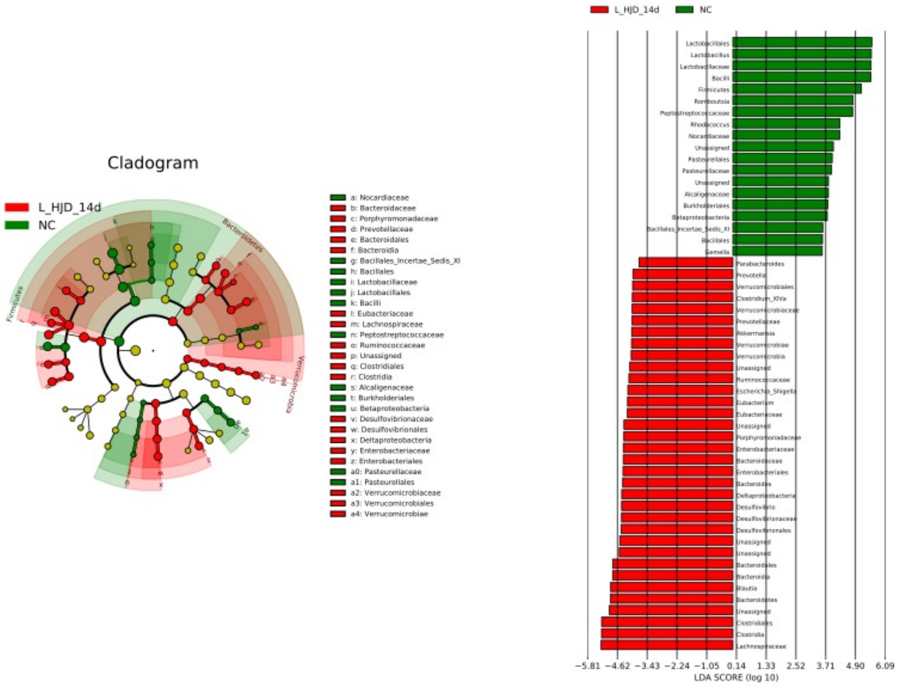

Figure S6

Taxonomic cladogram obtained by LEfSe. Differences are represented by the color of the most abundant class. Histogram of the linear discriminant analysis (LDA) score for the gut microbiota in the NC and L_HJD_14d of rats. The statistically significant differences in the microbiota of the two groups were analyzed using linear discriminant analysis effect size (LEfSe) statistical analysis for the identification of biomarkers between two groups and is shown in the histogram. The length of histogram represents the degree of influence of the different microbiota. Red indicating L_HJD_14d, green NC.

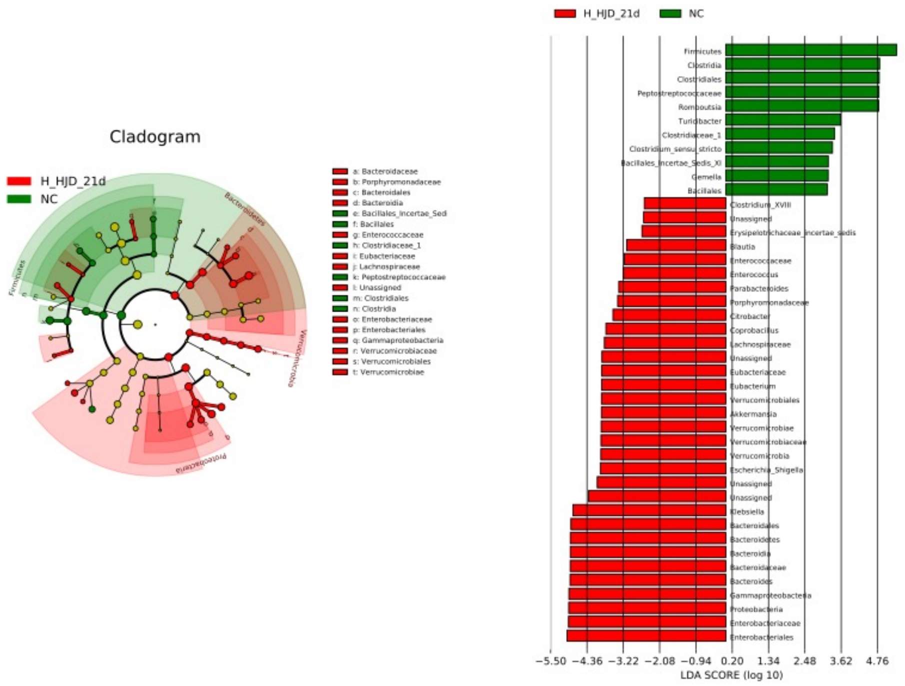

Figure S7

Taxonomic cladogram obtained by LEfSe. Differences are represented by the color of the most abundant class. Histogram of the linear discriminant analysis (LDA) score for the gut microbiota in the NC and H_HJD_21d of rats. The statistically significant differences in the microbiota of the two groups were analyzed using linear discriminant analysis effect size (LEfSe) statistical analysis for the identification of biomarkers between two groups and is shown in the histogram. The length of histogram represents the degree of influence of the different microbiota. Red indicating H_HJD_21d, green NC.

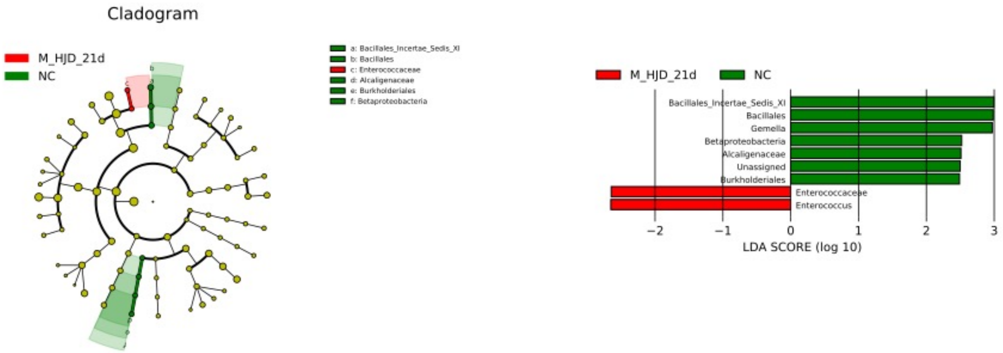

Figure S8

Taxonomic cladogram obtained by LEfSe. Differences are represented by the color of the most abundant class. Histogram of the linear discriminant analysis (LDA) score for the gut microbiota in the NC and H_HJD_21d of rats. The statistically significant differences in the microbiota of the two groups were analyzed using linear discriminant analysis effect size (LEfSe) statistical analysis for the identification of biomarkers between two groups and is shown in the histogram. The length of histogram represents the degree of influence of the different microbiota. Red indicating M_HJD_21d, green NC.

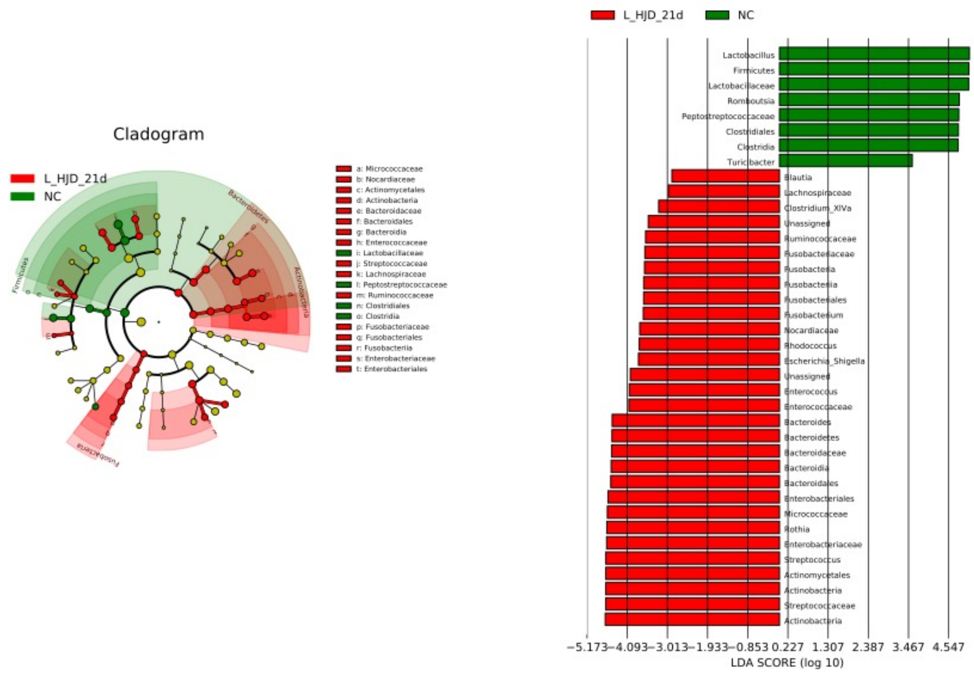

Figure S9

Taxonomic cladogram obtained by LEfSe. Differences are represented by the color of the most abundant class. Histogram of the linear discriminant analysis (LDA) score for the gut microbiota in the NC and L_HJD_21d of rats. The statistically significant differences in the microbiota of the two groups were analyzed using linear discriminant analysis effect size (LEfSe) statistical analysis for the identification of biomarkers between two groups and is shown in the histogram. The length of histogram represents the degree of influence of the different microbiota. Red indicating L_HJD_21d, green NC.
